# Effect of virtual reality-based training on postural balance in children and adolescents with neurodevelopmental disorders: a systematic review and meta-analysis

**DOI:** 10.3389/fpubh.2025.1719286

**Published:** 2026-01-09

**Authors:** Baifa Zhang, Yu Liu, Zhicheng Lin, Chao Li

**Affiliations:** 1 School of Sports, Southwest University, Chongqing, China; 2 School of Sports, Qingdao University, Qingdao, China

**Keywords:** adolescents, children, neurodevelopmental disorders, postural balance, virtual reality

## Abstract

**Purpose:**

Virtual reality, an emerging rehabilitation technology, is increasingly used to improve postural balance in children and adolescents with neurodevelopmental disorders. This review evaluated the effectiveness of virtual reality interventions for improving postural balance in this population based on randomized controlled trials.

**Materials and methods:**

PubMed, Web of Science, Scopus, Embase, Cochrane Library, CNKI, and Wanfang were systematically searched from database inception through November 6, 2025, for English- and Chinese-language randomized controlled trials examining virtual reality–based interventions on objective measures of postural balance in children and adolescents with neurodevelopmental disorders. Methodological quality was evaluated using the Cochrane Risk of Bias 2 tool, and the certainty of evidence was appraised according to the GRADE approach. Pooled standardized mean differences with 95% confidence intervals were computed using the meta package in R (version 4.5.1), and heterogeneity, subgroup, and leave-one-out sensitivity analyses were performed.

**Results:**

Ten RCTs involving 372 participants were included. Meta-analysis demonstrated that virtual reality–based interventions were associated with statistically significant improvements in postural balance compared with control conditions (SMD = 0.30, 95%CI [0.09, 0.51], *p* = 0.004). Although trials with shorter session durations, lower weekly training frequencies, and shorter intervention periods appeared to yield larger effects, subgroup analyses failed to identify statistically significant between-subgroup differences.

**Conclusion:**

This systematic review and meta-analysis indicates that virtual reality–based interventions may enhance postural balance in children and adolescents with neurodevelopmental disorders. However, the certainty of evidence remains limited owing to the paucity of included trials and methodological limitations. Future large-scale randomized controlled trials with extended follow-up periods and standardized balance assessments are warranted to corroborate these findings and optimize intervention parameters.

**Systematic review registration:**

https://www.crd.york.ac.uk/prospero/display_record.php?ID=CRD420251135718, CRD420251135718.

## 1 Introduction

Neurodevelopmental disorders constitute a heterogeneous group of conditions with early onset, characterized by atypical central nervous system development that results in impairments in cognitive, social, motor, academic, or occupational functioning (1, 2). These disorders encompass attention-deficit/hyperactivity disorder (ADHD), autism spectrum disorder (ASD), intellectual disability (ID), specific learning disorders (SLD), developmental coordination disorder (DCD), and genetic syndromes such as Down syndrome (DS) (3). The prevalence of neurodevelopmental disorders among children and adolescents has increased substantially over the past two decades, generating significant demand for educational and behavioral services (4, 5). Motor impairments such as clumsiness, postural instability, and motor coordination abnormalities are often the first signs of an increased risk of neurodevelopmental disorders in young children (6, 7). Among these motor deficits, postural control impairments are consistently observed in children and adolescents with neurodevelopmental disorders, particularly when vestibular function must compensate for absent or attenuated somatosensory and visual inputs (8–12). Postural control is a complex motor skill requiring the integration of multiple sensorimotor processes, including biomechanical constraints, movement strategies, sensory strategies, spatial orientation, dynamic control, and cognitive processing (13). Since postural control is essential for standing, walking, and daily functioning, deficits during critical developmental periods may contribute to the atypical motor and social skills characteristic of neurodevelopmental disorders (14).

Researchers have investigated various intervention approaches that demonstrate improvements in postural control impairments among children and adolescents with neurodevelopmental disorders. These interventions include movement-based activities (hippotherapy, aquatic therapy, martial arts, dance, skating, and structured physical activity programs), neuromodulation techniques (transcranial direct current stimulation), and conventional physical therapy approaches (balance training, neuromuscular stretching, and resistance training) (14–18). Neuroplasticity underlies functional reorganization of the central nervous system, indicating that effective postural control interventions require extensive repetition (19, 20). Moreover, research has also found that interventions have a greater effect on motivated individuals, thus interventions need to incorporate variability and motivational elements to ensure sufficient engagement (21, 22). Furthermore, evidence demonstrates enhanced intervention efficacy among motivated participants, and because children with neurodevelopmental disorders frequently exhibit limited social and physical engagement due to reduced enjoyment (23), interventions must incorporate motivational elements and variability to sustain participation (21, 22). The development of novel technologies for rehabilitation, such as virtual reality (VR), appears to have beneficial effects on postural control in children and adolescents with neurodevelopmental disorders (24, 25). VR integrates hardware and software systems to generate immersive digital environments that deliver multisensory feedback, including visual, auditory, proprioceptive, and tactile inputs, thereby enabling dynamic real-time interaction (26). Based on immersion and presence levels, virtual reality systems are classified as non-immersive (NIVR) or immersive (IVR). Virtual reality facilitates skill development through experiential learning in safe, gamified environments that enhance motivation, enjoyment, and self-competition, yielding improved performance outcomes (27–29).

Previous meta-analyses have suggested that virtual reality interventions may help to alleviate postural balance deficits in children and adolescents with various neurodevelopmental conditions, such as DS (24) and DCD (25). However, these meta-analyses typically examined individual neurodevelopmental disorders, thereby limiting comparisons of treatment effects across disorders. Therefore, this meta-analysis synthesizes current evidence to evaluate the effectiveness of VR interventions on postural balance across different neurodevelopmental disorders and elucidate their therapeutic potential in this population.

## 2 Materials and methods

This systematic review and meta-analysis adheres to the Preferred Reporting Items for Systematic Reviews and Meta-Analyses (PRISMA) reporting guidelines. The protocol was registered in PROSPERO (CRD420251135718).

### 2.1 Data source and search strategy

A comprehensive literature search was conducted in PubMed, Web of Science, Scopus, Embase, Cochrane Library, China National Knowledge Infrastructure (CNKI), and Wanfang databases from inception to November 6, 2025. The search was restricted to English- and Chinese-language peer-reviewed articles. The search strategy employed Medical Subject Headings (MeSH) including the following terms: child, adolescent, neurodevelopmental disorders, attention deficit disorder with hyperactivity, autism spectrum disorder, developmental disabilities, motor skills disorders, intellectual disability, virtual reality exposure therapy, virtual reality, exergaming, postural balance, and motor skills. Relevant synonyms were incorporated to ensure comprehensive retrieval across title, abstract, and keyword fields within the selected databases. In addition, we manually searched the reference lists of all included studies to identify further relevant articles. Complete search strategies for each database are provided in Table 1 (Supplementary material 1).

**Table 1:** Characteristics of the included studies.

Study & year	Country	Diagnosis	Age (years)	Sample size (IG/CG)	Dosage	VR intervention	Control	Measuring scale
Harkness-Armstrong et al., 2025 (36)	United Kingdom	DCD	8–10	11/6	30 min/d × 3 d/wk. × 6 wk	Nintendo Switch	No intervention	MABC-2
Yunus et al., 2024 (48)	Indonesia	DS	9–18	10/10	20 min/d × 2 d/wk. × 4 wk	VR SenMor	No intervention	PBS
Wang et al., 2022 (49)	China	DS	11–18	15/15	40 min/d × 3 d/wk. × 8 wk	Xbox Kinect	PE course	BBS
Rafiei Milajerdi et al., 2021 (51)	Iran	ASD	6–10	20/20	35 min/d × 3 d/wk. × 8 wk	Xbox Kinect	PE course	MABC-2
Cavalcante Neto et al., 2020 (42)	Brazil	DCD	7–10	16/16	60 min/d × 2 d/wk. × 8 wk	Nintendo Wii	TST	MABC-2
Bonney et al., 2017 (43)	South Africa	DCD	13–16	22/21	45 min/d × 1 d/wk. × 14 wk	Nintendo Wii	TFT	MABC-2
Hammond et al., 2014 (44)	United Kingdom	DCD	7–10	10/8	10 min/d × 3 d/wk. × 4 wk	Nintendo Wii	PE course	BOT-2
Mombarg et al., 2013 (45)	Netherlands	DCD	7–12	15/14	10 min/d × 3 d/wk. × 4 wk	Nintendo Wii	No intervention	MABC-2
Salem et al., 2012 (47)	United States	DCD	3–5	20/20	30 min/d × 3 d/wk. × 6 wk	Nintendo Wii	Traditional rehabilitation	GMFM-88
Wuang et al., 2011 (50)	Taiwan, China	DS	7–12	52/53	60 min/d × 2 d/wk. × 24 wk	Nintendo Wii	No intervention	BOT

DCD, Developmental Coordination Disorder; DS, Down Syndrome; ASD, Autism Spectrum Disorder; DSM-5, Diagnostic and Statistical Manual of Mental Disorders, Fifth Edition; ADOS-2, Autism Diagnostic Observation Schedule, Second Edition; DCDQ, Developmental Coordination Disorder Questionnaire; MABC-2, Movement Assessment Battery for Children-2; BOT-2, Bruininks–Oseretsky Test of Motor Proficiency-2; PBS, Pediatric Balance Scale; BBS, Berg Balance Scale; GMFM-88, Gross Motor Function Measure-88; PE, Physical education, TST, Task-specific matched training; TFT, Task-oriented functional training.

### 2.2 Identifying relevant studies

Three reviewers used the Cochrane PICOS (Population, Intervention, Comparison, Outcomes, and Study design) framework to establish eligibility criteria. Eligibility criteria were as follows: (1) participants (P): children and adolescents aged 2–18 years with neurodevelopmental disorders (e.g., DCD, ASD, ADHD, ID); (2) interventions (I): VR-based therapeutic approaches; (3) control (C) groups: active control group (e.g., physical education) or passive control group (e.g., no intervention); (4) outcomes (O): predefined objective outcome measures for assessing balance; (5) study (S) designs: RCTs. Studies were excluded if they did not meet the eligibility criteria, were narrative, systematic, or scoping reviews, involved infants, had outcome indicators that did not meet analysis requirements, or had missing data. After conducting comprehensive database searches, all identified studies were recorded and duplicates were removed. Two reviewers independently screened relevant studies by reviewing titles, abstracts, and full texts. Discrepancies between the two reviewers were resolved through discussion with a third reviewer.

### 2.3 Data extraction

Two reviewers independently extracted data from all included studies using a standardized form, collecting variables that included study characteristics (author, year of publication, and country), participant information (disease type, diagnostic method, age range, and sample sizes), intervention details (VR platform, session duration, weekly frequency, and total intervention period), control conditions, and outcome measures (assessment instruments) (30, 31). Any discrepancies or divergences in data extraction were resolved through discussion between the two reviewers to reach consensus, with a third reviewer making the final decision when agreement could not be achieved.

### 2.4 Risk of bias and certainty of evidence

Risk of bias in included studies was assessed using the Cochrane Risk of Bias 2 (RoB 2) tool, which evaluates five domains: bias arising from the randomization process, deviations from intended interventions, missing outcome data, outcome measurement, and selection of reported results, culminating in an overall risk-of-bias judgment (32). Two reviewers independently conducted assessments. Disagreements were resolved through consensus or third reviewer arbitration. Publication bias was assessed using visual inspection of funnel plots and Egger’s test. Publication bias was assessed using visual inspection of funnel plots and Egger’s test. Two independent reviewers assessed the certainty of evidence using the Grading of Recommendations, Assessment, Development, and Evaluation (GRADE) framework, which categorizes evidence as very low, low, moderate, or high (33). Because all included studies were RCTs, evidence started at high certainty. This rating was subsequently downgraded when concerns were identified regarding risk of bias, inconsistency, indirectness, imprecision, or publication bias (34, 35).

### 2.5 Data synthesis

All statistical analyses were performed using R software (version 4.5.1) with the meta packages. Standardized mean differences (SMDs) with 95% confidence intervals (CIs) were calculated as the summary measure for continuous variables. Heterogeneity was subsequently assessed using Cochran’s Q test and the I^2^ statistic, with a fixed-effects model applied when heterogeneity was low (*p* > 0.10 and I^2^ < 50%) and a random-effects model employed otherwise (36). Additionally, sensitivity analysis was conducted using a leave-one-out approach to assess the influence of individual studies on the pooled estimate. Subgroup analyses were then performed based on VR intervention features (duration, frequency, and period) (37–40), control group types (31), and participant characteristics (diagnosis) (41).

## 3 Results

### 3.1 Study selection

**Figure 1:**
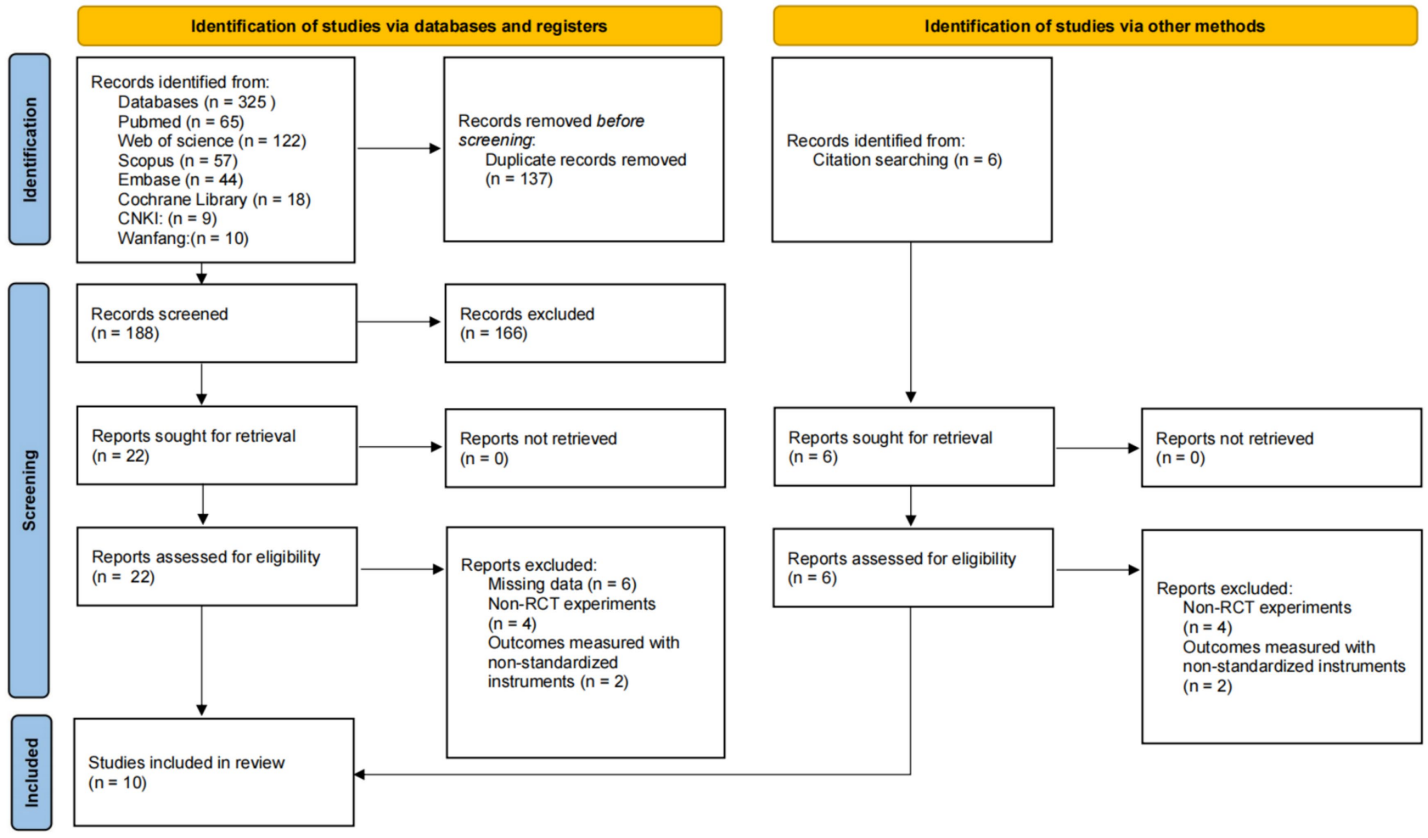
PRISMA flow diagram of selected studies.

The study selection process is illustrated in Figure 1. A comprehensive search of seven databases yielded 325 records: PubMed (*n* = 65), Web of Science (*n* = 122), Scopus (*n* = 57), Embase (*n* = 44), Cochrane Library (*n* = 18), CNKI (*n* = 9), and Wanfang (*n* = 10). Following the removal of 137 duplicates, 188 studies were screened. After preliminary screening, 22 articles underwent full-text review, of which 10 met the final inclusion criteria. Additionally, 6 records identified through manual search were excluded as they did not meet the inclusion criteria.

### 3.2 Study characteristics

The included RCT studies, published between 2011 and 2025, comprised 372 children and adolescents aged 3–18 years with neurodevelopmental disorders, with 191 participants allocated to intervention groups and 183 to control groups. The specific conditions represented were DCD (*n* = 6) (42–47), DS (*n* = 3) (48–50), ASD (*n* = 1) (51). The interventions predominantly utilized non-immersive VR training through commercial motion-controlled gaming systems, comprising Nintendo Wii (*n* = 6) (42–45, 47, 50), Xbox Kinect (*n* = 2) (49, 51), and Nintendo Switch (*n* = 1) (46). In contrast, one study implemented VR SenMor, an immersive, custom-developed sensorimotor VR platform that delivered gamified balance training via a head-mounted display (48). Intervention sessions ranged from 10 to 60 min in duration, occurred one to three times weekly, and extended over 4 to 24 weeks. Meanwhile, control groups consisted of passive controls receiving no intervention (*n* = 4) (45, 46, 48, 50) and active controls receiving physical education (PE; *n* = 3) (44, 49, 51), task-specific matched training (TST; *n* = 1) (42), task-oriented functional training (TFT; *n* = 1) (43), or traditional rehabilitation (*n* = 1) (47). To evaluate the effects of virtual reality-based training on postural balance, researchers utilized validated assessment tools: the Movement Assessment Battery for Children-2 (MABC-2; *n* = 5) (42, 43, 45, 46, 51), the Bruininks–Oseretsky Test of Motor Proficiency-2 (BOT-2; *n* = 2) (44, 50), the Pediatric Balance Scale (PBS; *n* = 1) (48), the Berg Balance Scale (BBS; *n* = 1) (49), and the Gross Motor Function Measure-88 (GMFM-88; *n* = 1) (47). Table 1 presents the characteristics of the included RCTs.

### 3.3 Risk of bias and certainty of evidence

**Figure 2:**
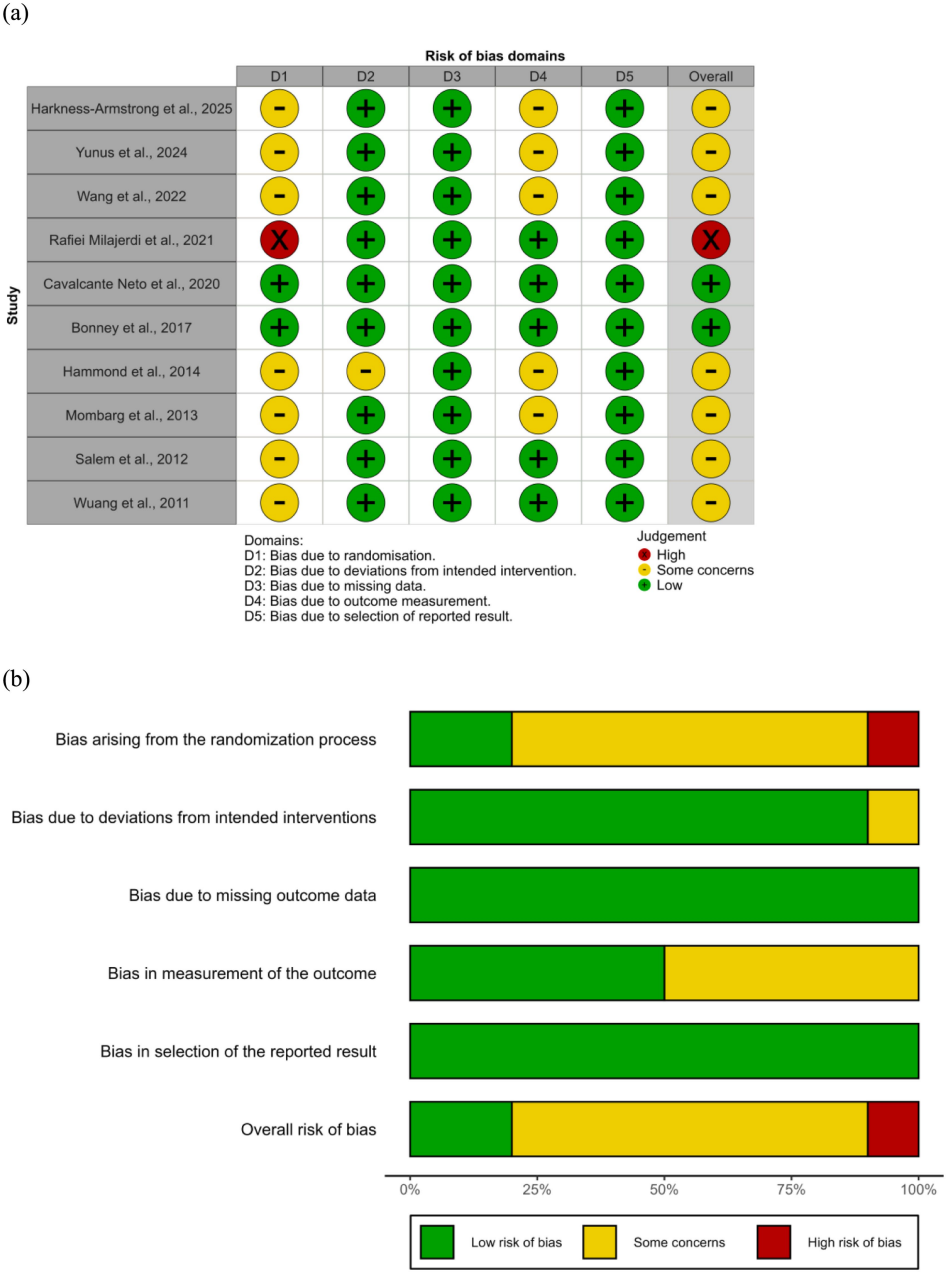
Risk of bias assessment: **(a)** Summary plot and **(b)** traffic light plot.

**Figure 3:**
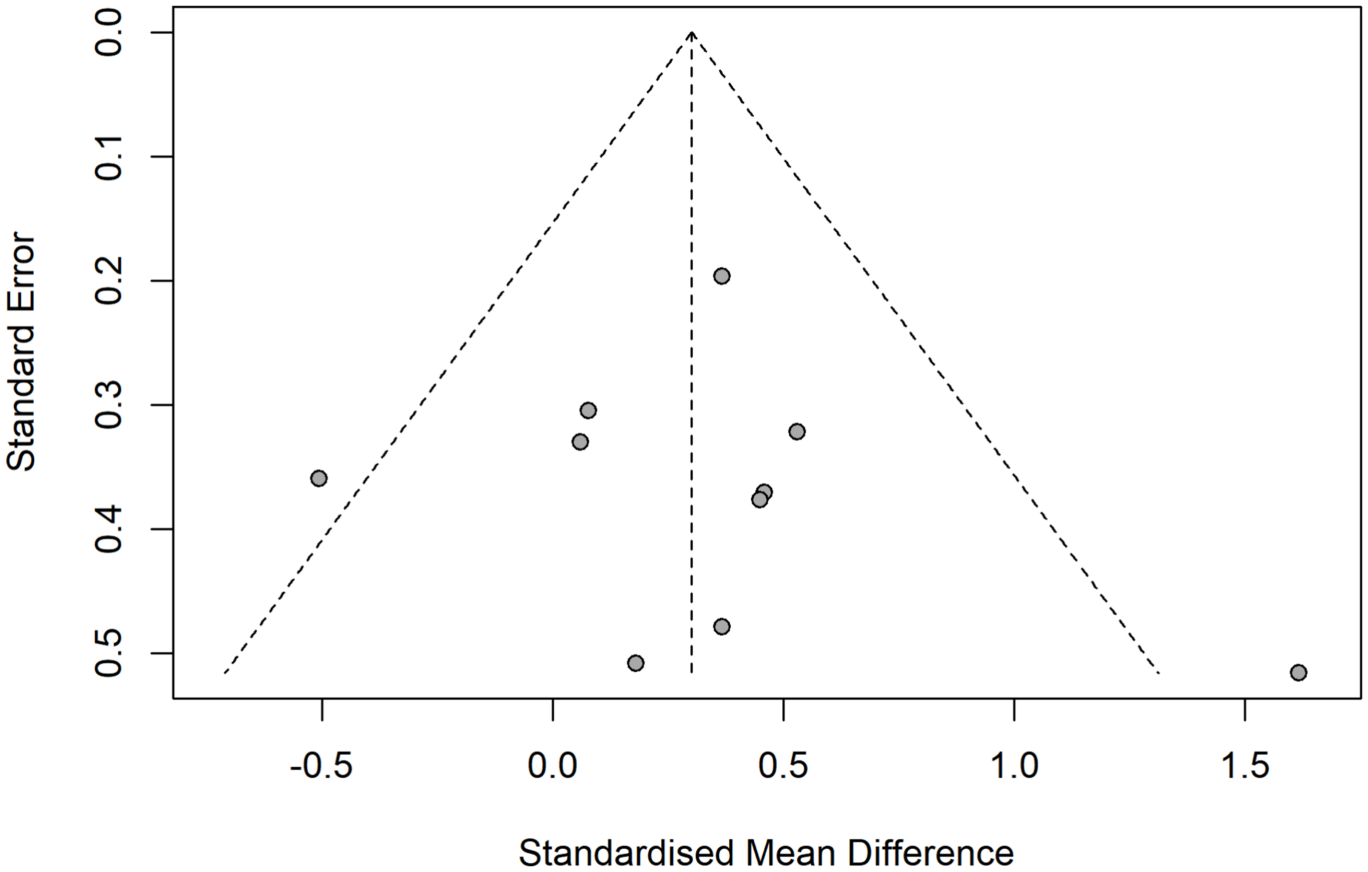
Funnel plot of publication bias in VR-based interventions on postural balance in children with neurodevelopmental disorders.

The methodological quality of the included studies was assessed using the RoB 2. The majority of trials demonstrated acceptable methodological rigor, though most were assessed as having some concerns regarding bias, with only one study (51) rated as high risk overall. Across domains, the risk of bias arising from deviations from intended interventions, missing outcome data, and selective reporting was generally low. In contrast, concerns were more frequent regarding randomization procedures and outcome measurement, as several studies lacked sufficient detail on allocation concealment (44–50) or assessor blinding (44–46, 48, 49). Figure 2 summarizes the domain-specific ratings. Visual inspection of the funnel plot (Figure 3) revealed approximate symmetry, with effect estimates clustered near the pooled standardized mean difference and no apparent small-study effects. Egger’s regression test confirmed this observation, showing no significant asymmetry (intercept = 0.72, 95% CI − 2.02 to 3.46, *p* = 0.621). Table 2 presents the GRADE assessment results. Evidence certainty was rated as moderate across all 10 included RCTs due to concerns about bias.

**Table 2:** GRADE assessment for the certainty of evidence.

Certainty of evidence							No. of patients		Effect		Certainty	Importance
No. of studies	Study design	Risk assessment	Inconsistency	Indirect evidence	Vagueness	Publication bias	Intervention	Comparison	Relative (95% CI)	Absolute (95% CI)		
10	RCT	Serious	It is not serious	It is not serious	It is not serious	It is not serious	189/372 (50.8%)	183/372 (49.2%)	Not estimable		+++ Moderate	IMPORTANT

### 3.4 Meta-analysis results

**Figure 4:**
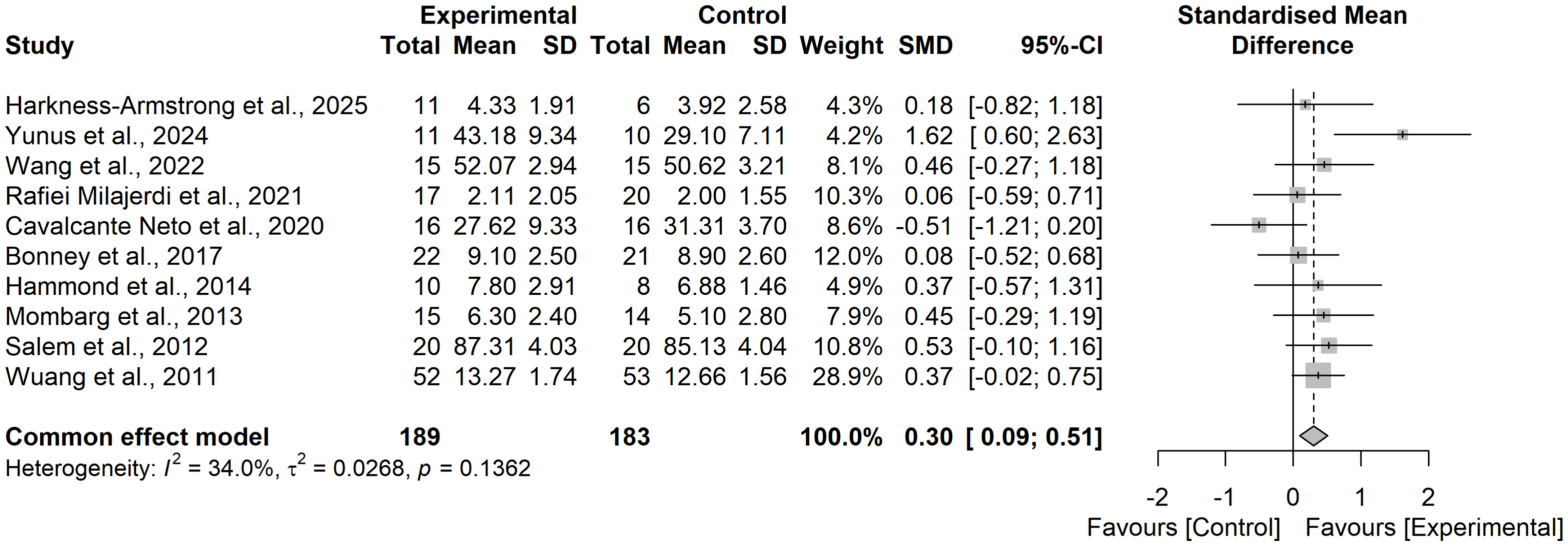
Forest plot of the effects of VR-based interventions on postural balance in children with neurodevelopmental disorders.

**Figure 5:**
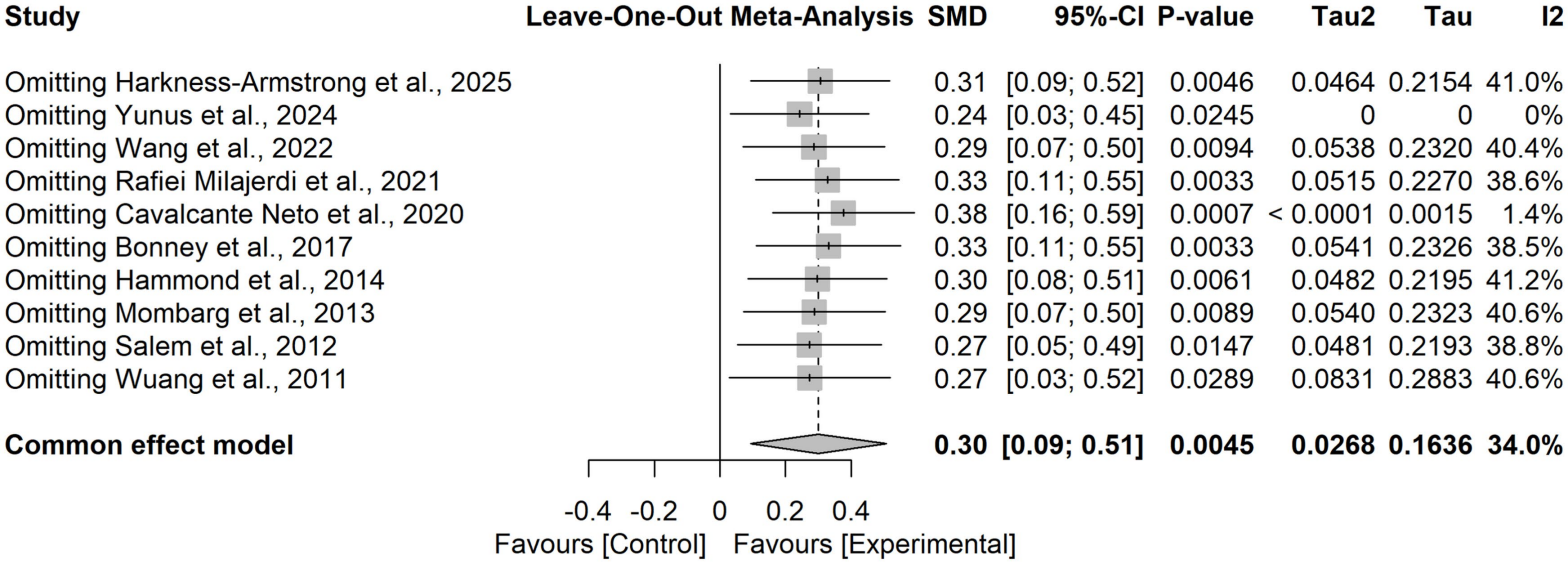
Leave-one-out sensitivity analysis for the effect of VR-based interventions on postural balance in children with neurodevelopmental disorders.

Pooled results from 10 RCTs demonstrated that virtual reality based training significantly improved postural balance in children and adolescents with neurodevelopmental disorders compared with control interventions (SMD = 0.30, 95% CI = 0.09 to 0.51). The heterogeneity test indicated low-to-moderate heterogeneity (I^2^ = 34.0%, τ^2^ = 0.0268, *p* = 0.1362), and thus a fixed-effects model was applied. Sensitivity analysis using a leave-one-out approach confirmed the robustness of the findings (Figure 4), with pooled SMD values ranging from 0.24 to 0.38 and all models remaining statistically significant. Notably, when the study by Yunus et al. (48) was excluded, heterogeneity was reduced to I^2^ = 0%, suggesting that this trial was the primary source of heterogeneity. This study uniquely employed an immersive VR intervention, whereas the other trials predominantly used non-immersive VR approaches (Figure 5).

**Figure 6:**
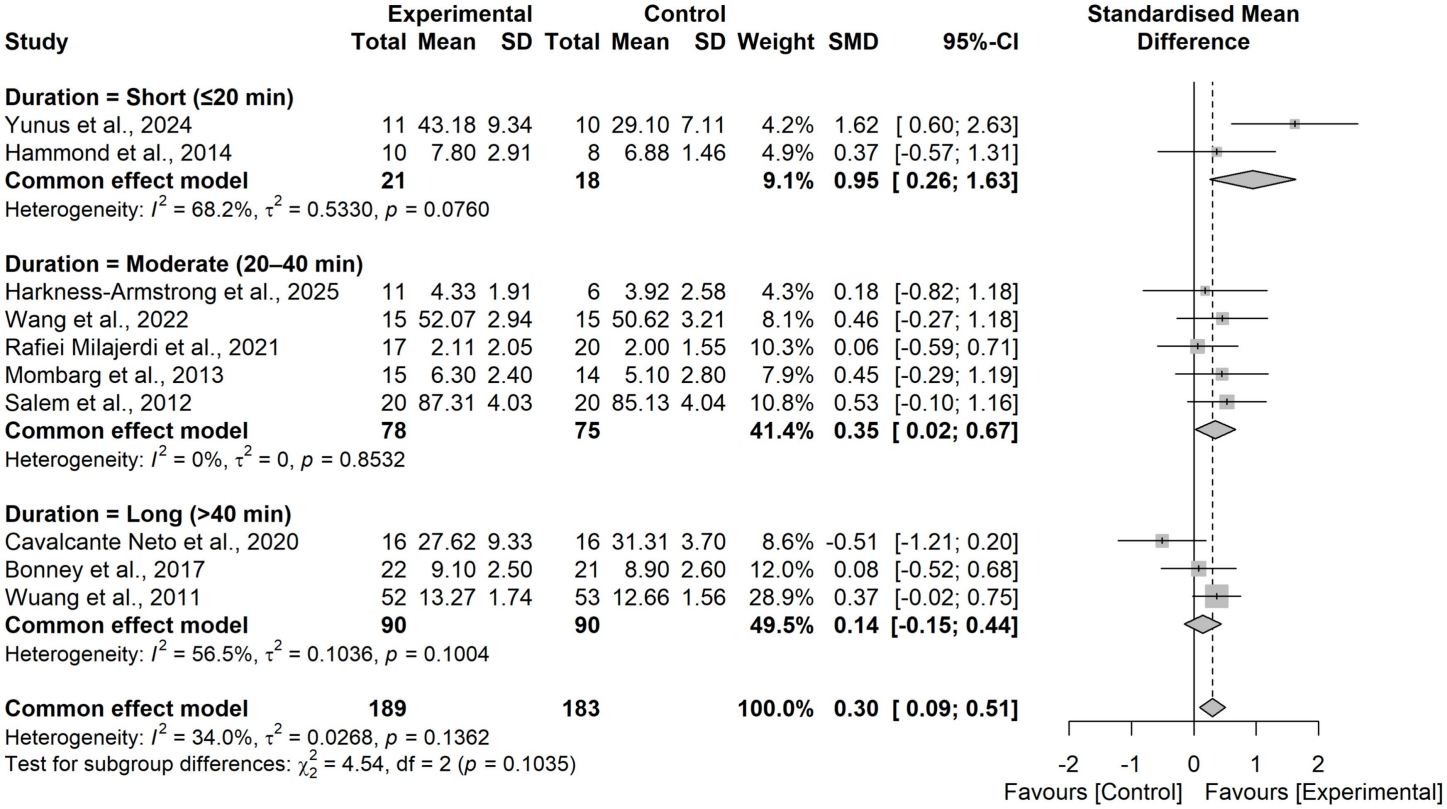
Forest plot for subgroup analysis according to the intervention session duration.

In the subgroup analysis by session duration, significant effects were observed for short sessions (≤20 min; SMD = 0.95, 95% CI 0.26–1.63) and moderate sessions (20–40 min; SMD = 0.35, 95% CI 0.02–0.67), whereas long sessions (>40 min) yielded a small, non-significant effect (SMD = 0.14, 95% CI − 0.15–0.44). The between-group difference was not significant (χ^2^₂ = 4.54, *p* = 0.10; Figure 6).

In the subgroup analysis by training frequency, a significant effect was observed for low-frequency protocols (1–2 sessions/week; SMD = 0.30, 95% CI 0.05–0.56), whereas high-frequency protocols (3–4 sessions/week) yielded a non-significant effect (SMD = 0.29, 95% CI − 0.05–0.64). The between-group difference was not significant (χ^2^₁ = 0.00, *p* = 0.96).

**Figure 7:**
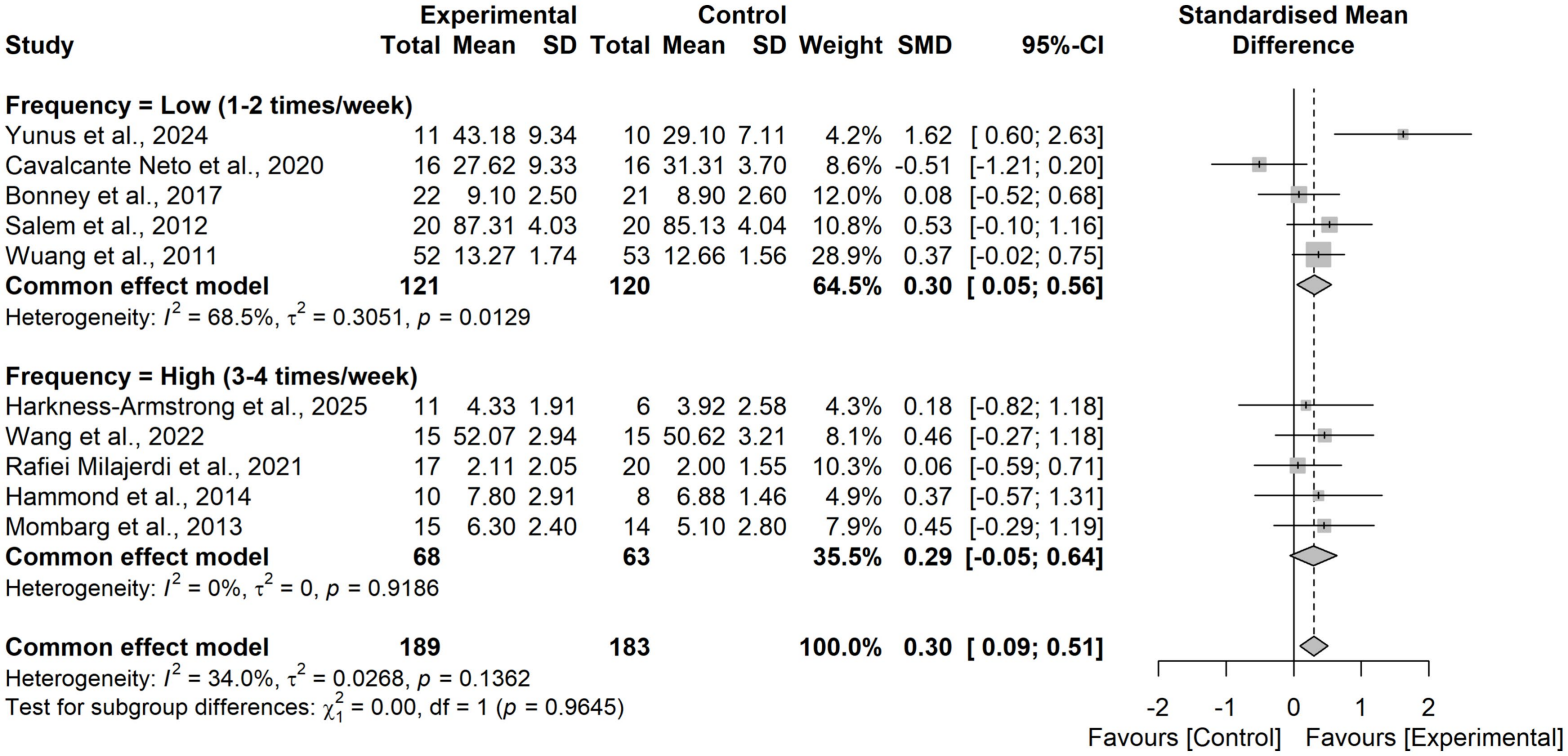
Forest plot for subgroup analysis according to the intervention frequency.

In the subgroup analysis by intervention period, short-term programs (≤6 weeks) demonstrated a moderate effect on balance (SMD = 0.61, 95% CI 0.16–1.06), whereas longer programs (>6 weeks) yielded a smaller, non-significant effect (SMD = 0.22, 95% CI − 0.02–0.45). The between-group difference was not significant (χ^2^₁ = 2.25, *p* = 0.13; Figure 7).

**Figure 8:**
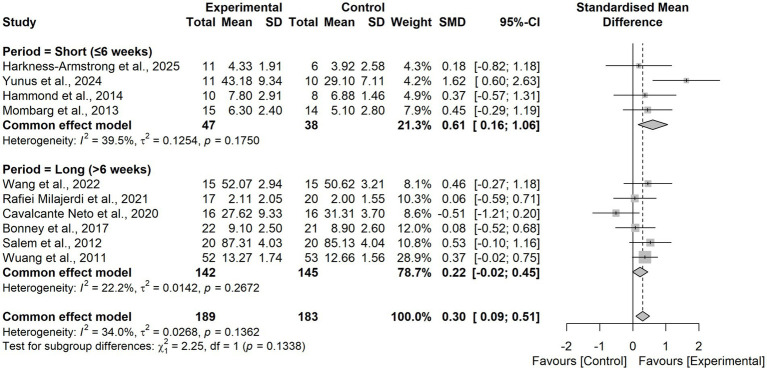
Forest plot for subgroup analysis according to the intervention period.

Subgroup analysis by diagnosis type revealed no statistically significant difference (χ^2^₂ = 2.89, *p* = 0.24; Figure 8).

**Figure 9:**
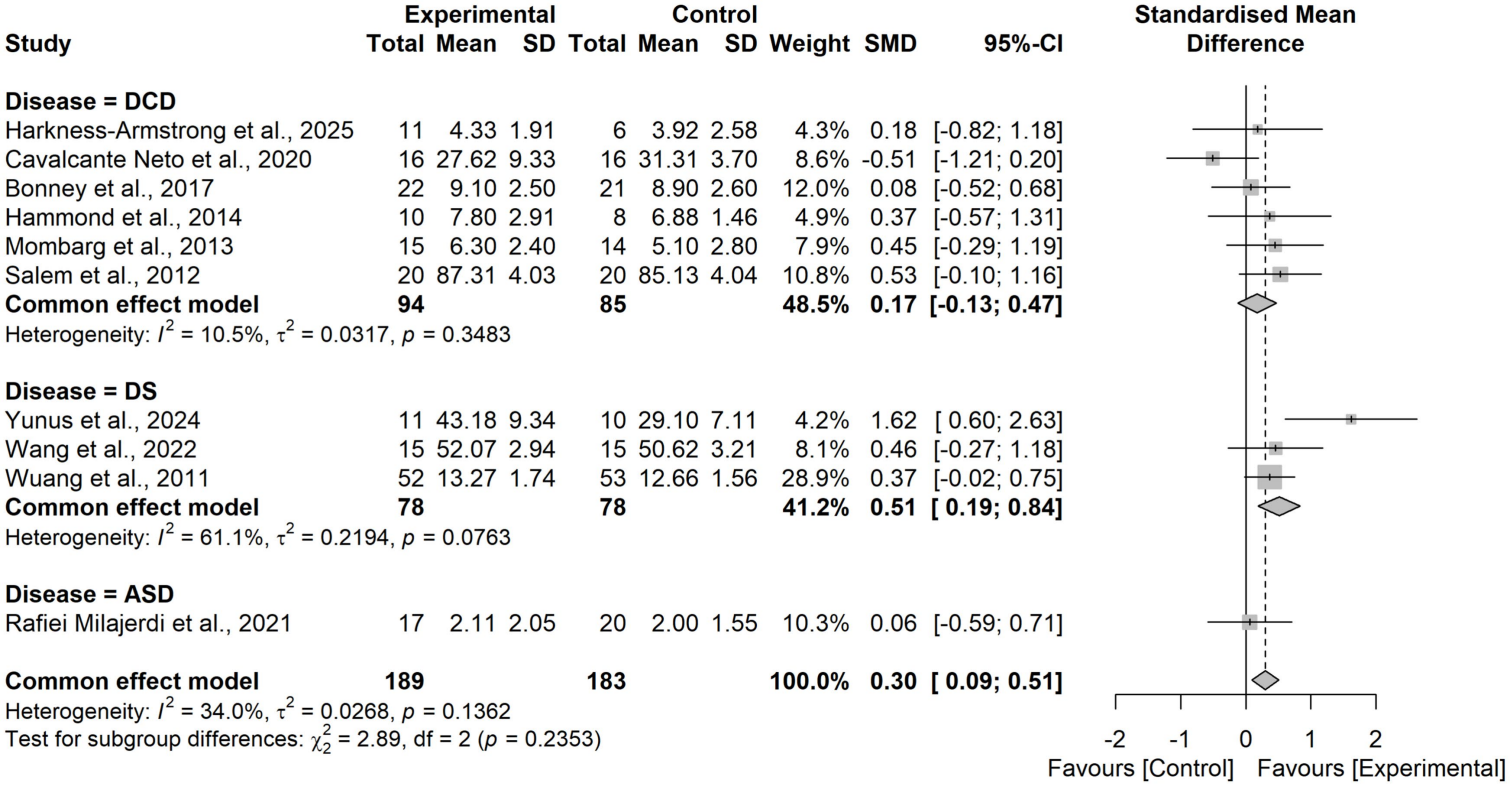
Forest plot for subgroup analysis according to the diagnosis.

**Figure 10:**
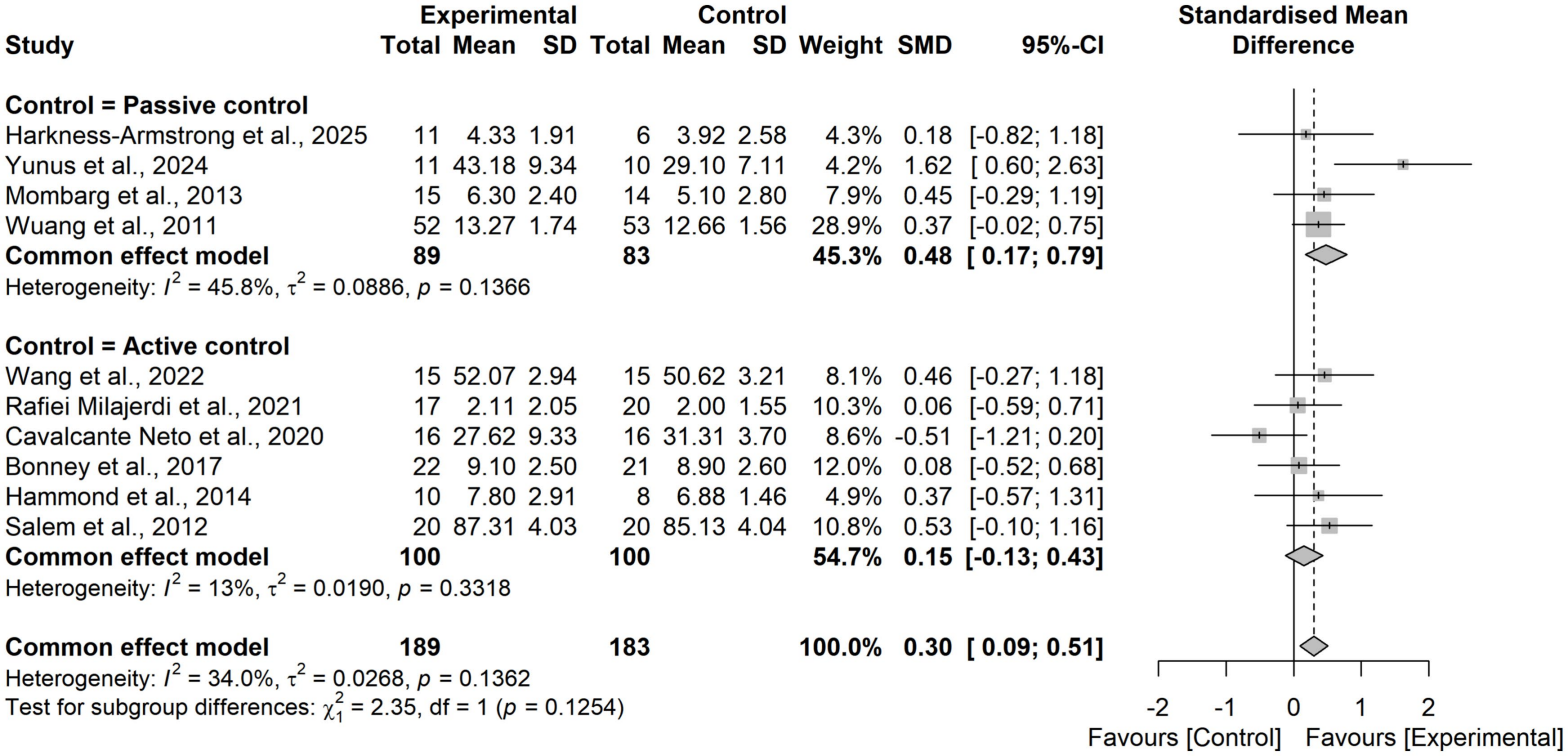
Forest plot for subgroup analysis according to control group types.

Subgroup analysis by control group type revealed no statistically significant difference (χ^2^₁ = 2.35, *p* = 0.13; Figures 9, 10).

## 4 Discussion

This systematic review and meta-analysis examined 10 randomized controlled trials (n = 372) to evaluate the effectiveness of virtual reality interventions on postural balance in children and adolescents with neurodevelopmental disorders. Balance outcomes were assessed using standardized instruments, including the Movement Assessment Battery for Children-2 (MABC-2), Bruininks–Oseretsky Test of Motor Proficiency-2 (BOT-2), Pediatric Balance Scale (PBS), Berg Balance Scale (BBS), and Gross Motor Function Measure-88 (GMFM-88). The included interventions predominantly employed non-immersive virtual reality devices. The meta-analysis revealed that virtual reality-based interventions were more effective than conventional therapy for improving postural balance outcomes (SMD = 0.30). Although subgroup analyses revealed no statistically significant differences for any examined moderator, a trend toward greater benefit was observed in trials with shorter sessions (≤20 min or 20–40 min), lower training frequencies (1–2 sessions/week), and shorter intervention durations (≤6 weeks).

Our meta-analysis revealed a moderate effect of virtual reality-based interventions on postural balance outcomes in children and adolescents with neurodevelopmental disorders compared with conventional therapy, as interpreted according to the revised benchmarks for effect size in rehabilitation research, which may suggest possible clinical relevance of the finding (52). Given the limited number of included studies, the clinical significance of the finding for postural balance warrants confirmation through future research with extended follow-up. Although incompletely understood, virtual reality likely improves postural balance through neuroplastic adaptations, including neural pathway formation and mirror neuron activation that facilitate imitative learning (53). From the perspective of motor learning, extensive practice drives neuroplastic changes that underlie motor skill acquisition (54). Interactive gaming features in virtual reality environments enable extensive task repetition while reducing fatigue awareness. For example, several studies (42–45, 47) in this review utilizing the Wii Balance Board reported positive balance outcomes, potentially due to games (e.g., snowboard slalom) that induce repeated lateral weight shifts reinforced by motivational elements including point-based systems and unlockable content. Moreover, implementing multiple virtual reality game variations mitigates motivation decline from habituation to repetitive gameplay (55). Mombarg et al. selected 3–5 games per session to ensure adequate training variety while automatically adjusting difficulty levels based on skill progression evidenced by performance improvements. Virtual reality game-based interventions enhance motor development in children and adolescents with neurodevelopmental disorders by providing diverse movement experiences, thereby promoting skill generalization across multiple task contexts (56). Furthermore, real-time augmented feedback delivered through visual display systems constitutes a form of implicit learning that enables individuals to refine motor skills through enhanced visual and somatosensory control, thereby promoting neuroplastic reorganization of sensorimotor circuits (50, 57) (e.g., hitting targets) rather than internal body movements aligns with Wulf and Lewthwaite’s model of motor learning, whereby externally focused practice facilitates implicit skill acquisition that demonstrates greater resilience under stress and enhanced integration with existing motor competencies. Children’s attentional focus on external cues (e.g., hitting targets) rather than internal body movements aligns with Wulf and Lewthwaite’s model of motor learning, whereby externally focused practice facilitates implicit skill acquisition that demonstrates greater resilience under stress and enhanced integration with existing motor competencies (58, 59).

The type of VR device (immersive versus non-immersive) was identified as the primary source of heterogeneity in our meta-analysis, with the interventions predominantly using non-immersive VR training through commercial motion-controlled gaming systems. Non-immersive VR provides practical advantages, including lower cost, portability, and ease of use, while minimizing adverse events such as cybersickness and visual disturbances commonly associated with immersive systems (60). Various commercial gaming platforms employ different motion capture technologies, including camera-based tracking (e.g., Microsoft Kinect), controller-based motion sensing with balance boards (e.g., Nintendo Wii), and handheld motion controllers with integrated accelerometers (e.g., Nintendo Switch). In the context of limited immersive VR research, Yunus et al. implemented VR SenMor, a custom-developed sensorimotor platform that provides 360° environmental visualization through head-mounted displays, delivering immersive experiences. Yunus et al. reported that the VR SenMor intervention demonstrated large effect sizes (d ≥ 0.8), exceeding those of other studies in this meta-analysis. The paucity of RCTs examining immersive VR interventions precludes robust comparisons of non-immersive versus immersive VR effectiveness in children and adolescents with neurodevelopmental disorders. In our meta-analysis, trials using shorter sessions, lower training frequencies, and shorter intervention periods tended to show larger effects than their higher-dose counterparts. However, the differences between these dose-related subgroups were not significant. These findings contrast with those of Rafiei Milajerdi et al., who proposed that VR interventions exceeding 16 h may be necessary to observe balance improvements due to a dose–response effect. From a neuroplasticity perspective, children efficiently develop novel cortical configurations even within brief training periods (61). Relatedly, short-term VR training has been shown to induce morphological changes in healthy adults, particularly in frontal and parietal gray matter and the right cerebellum, suggesting that brief VR exposure may suffice to elicit neural adaptations across diverse populations (62). Additionally, lower-dose interventions may preserve novelty and motivation, thereby enhancing adherence and engagement while mitigating the cognitive and physical fatigue associated with more intensive training (39). Consistent with motor learning principles suggesting that initial training phases yield greater gains, the observed trend may reflect rapid neuromotor acquisition following early exposure to VR-based stimuli (63).

## 5 Limitations

This study has limitations that should be considered when interpreting the findings. First, although the included studies focused on children and adolescents with neurodevelopmental disorders, these conditions are characterized by etiological and pathophysiological heterogeneity, resulting in diverse intervention requirements that may limit the generalizability of conclusions across populations (64). Second, the heterogeneity of interventions, encompassing varied VR platforms, game types, interaction modalities, and sensory feedback mechanisms, constrained the generalizability of findings. Moreover, given that only one study employed immersive VR devices, the effects of immersive VR on postural balance warrant further investigation. Third, the diversity of instruments used to assess postural balance yielded heterogeneous data across studies, complicating direct comparisons (65). Finally, the absence of large-sample, long-term randomized controlled trials, coupled with inconsistent protocols and control conditions in certain studies, precludes definitive conclusions and limits the generalizability of findings. Future research should incorporate extended follow-up periods to evaluate the durability of therapeutic gains from VR interventions. Moreover, future research should focus on determining optimal intervention formats and dosages, standardizing postural balance assessments, and assessing the effects of immersive VR on therapeutic outcomes.

## 6 Conclusion

This systematic review and meta-analysis indicates that VR-based interventions may enhance postural balance in children and adolescents with neurodevelopmental disorders. However, the certainty of evidence remains limited owing to the paucity of included studies and methodological limitations. Subgroup analyses revealed no statistically significant between-subgroup differences irrespective of session duration, training frequency, intervention period, diagnosis type, or control group type. Future research should employ extended follow-up periods to assess the durability of therapeutic gains, determine optimal intervention parameters, standardize postural balance assessments, and investigate the efficacy of immersive VR technologies.

## Data Availability

The original contributions presented in the study are included in the article/Supplementary material, further inquiries can be directed to the corresponding author.

## References

[1] Nisar S Haris M Fakhro KA. Genes and specific (related) proteins in neurodevelopmental disorders In: Qoronfleh MW Essa MM Saravana Babu C, editors. Proteins associated with neurodevelopmental disorders. Singapore: Springer Singapore (2022). 49–89.

[2] American Psychiatric Association. Diagnostic and statistical manual of mental disorders: DSM-5-TR. 5th ed., text revision ed. Washington, DC: American Psychiatric Publishing (2022).

[3] Bruni O Breda M Mammarella V Mogavero MP Ferri R. Sleep and circadian disturbances in children with neurodevelopmental disorders. Nat Rev Neurol. (2025) 21:103–20. doi: 10.1038/s41582-024-01052-9, 39779841

[4] Olusanya BO Smythe T Ogbo FA Nair MKC Scher M Davis AC. Global prevalence of developmental disabilities in children and adolescents: a systematic umbrella review. Front Public Health. (2023) 11:2009. doi: 10.3389/fpubh.2023.1122009, 36891340 PMC9987263

[5] Zablotsky B Black LI Blumberg SJ. Estimated prevalence of children with diagnosed developmental disabilities in the United States, 2014–2016. Hyattsville, MD: National Center for Health Statistics, Centers for Disease Control and Prevention (2017). 29235982

[6] Kangarani-Farahani M Malik MA Zwicker JG. Motor impairments in children with autism spectrum disorder: A systematic review and meta-analysis. J Autism Dev Disord. (2024) 54:1977–97. doi: 10.1007/s10803-023-05948-1, 36949273

[7] Meredith RM. Sensitive and critical periods during neurotypical and aberrant neurodevelopment: A framework for neurodevelopmental disorders. Neurosci Biobehav Rev. (2015) 50:180–8. doi: 10.1016/j.neubiorev.2014.12.001, 25496903

[8] Tsai C-L Wu SK Huang C-H. Static balance in children with developmental coordination disorder. Hum Mov Sci. (2008) 27:142–53. doi: 10.1016/j.humov.2007.08.002, 17935809

[9] Bucci MP Stordeur C Acquaviva E Peyre H Delorme R. Postural instability in children with ADHD is improved by methylphenidate. Front Neurosci. (2016) 10:163. doi: 10.3389/fnins.2016.00163, 27199629 PMC4854903

[10] Blomqvist S Olsson J Wallin L Wester A Rehn B. Adolescents with intellectual disability have reduced postural balance and muscle performance in trunk and lower limbs compared to peers without intellectual disability. Res Dev Disabil. (2013) 34:198–206. doi: 10.1016/j.ridd.2012.07.008, 22944259

[11] Enkelaar L Smulders E de Van Schrojenstein Lantman- Valk H Geurts ACH Weerdesteyn V. A review of balance and gait capacities in relation to falls in persons with intellectual disability. Res Dev Disabil. (2012) 33:291–306. doi: 10.1016/j.ridd.2011.08.028 22018534

[12] Fournier KA Kimberg CI Radonovich KJ Tillman MD Chow JW Lewis MH . Decreased static and dynamic postural control in children with autism spectrum disorders. Gait Posture. (2010) 32:6–9. doi: 10.1016/j.gaitpost.2010.02.007, 20400311 PMC2919314

[13] Lim YH Partridge K Girdler S. Standing postural control in individuals with autism spectrum disorder: systematic review and meta-analysis. J Autism Dev Disord. (2017) 47:144. doi: 10.1007/s10803-017-3144-y 28508177

[14] Date S Munn E Frey GC. Postural balance control interventions in autism spectrum disorder (ASD): a systematic review. Gait Posture. (2024) 109:170–82. doi: 10.1016/j.gaitpost.2024.01.034 38320424

[15] Shariat A Najafabadi MG Anastasio AT. The effectiveness of aquatic therapy on motor and social skill as well as executive function in children with neurodevelopmental disorder: a systematic review and meta-analysis. Arch Phys Med Rehabil. (2024) 105:025. doi: 10.1016/j.apmr.2023.08.025 37690741

[16] Kachouri H Borji R Baccouch R Laatar R Rebai H Sahli S. The effect of a combined strength and proprioceptive training on muscle strength and postural balance in boys with intellectual disability: an exploratory study. Res Dev Disabil. (2016) 53-54:367–76. doi: 10.1016/j.ridd.2016.03.003, 26994823

[17] Lee K Lee M Song C. Balance training improves postural balance, gait, and functional strength in adolescents with intellectual disabilities: single-blinded, randomized clinical trial. Disabil Health J. (2016) 9:416–22. doi: 10.1016/j.dhjo.2016.01.010, 26975417

[18] Gupta S Rao BK Kumaran S. Effect of strength and balance training in children with down’s syndrome: A randomized controlled trial. Clin Rehabil. (2011) 25:425–32. doi: 10.1177/0269215510382929, 21059663

[19] Johnston MV. Plasticity in the developing brain: implications for rehabilitation. Dev Disabil Res Rev. (2009) 15:94–101. doi: 10.1002/ddrr.64, 19489084

[20] Valvano J. Activity-focused motor interventions for children with neurological conditions. Phys Occup Ther Pediatr. (2004) 24:79–107. doi: 10.1300/J006v24n01_04, 15268999

[21] Purcell C Schott N Rapos V Zwicker JG Wilmut K. Understanding factors that influence physical activity behavior in people with developmental coordination disorder (DCD): A mixed-methods convergent integrated systematic review. Front Hum Neurosci. (2023) 17:1274510. doi: 10.3389/fnhum.2023.1274510, 38152480 PMC10751368

[22] Kwan MYW Li Y-C Cairney J. Theory-based correlates of physical activity among children with developmental coordination disorder: a scoping review. Curr Dev Disord Rep. (2022) 9:105–9. doi: 10.1007/s40474-022-00254-4

[23] Gobbi E Greguol M Carraro A. Brief report: exploring the benefits of a peer-tutored physical education programme among high school students with intellectual disability. J Appl Res Intellect Disabil. (2018) 31:937–41. doi: 10.1111/jar.12437, 29380486

[24] Piñar-Lara M Cortés-Pérez I Díaz-Fernández Á. Virtual reality-based therapy can enhance balance and muscular endurance in children and adolescents with down syndrome: a systematic review with a meta-analysis. Bioeng Basel Switz. (2024) 11:01–18. doi: 10.3390/bioengineering11111112, 39593772 PMC11591943

[25] Piñar-Lara M Obrero-Gaitán E Lomas-Vega R López-Ruiz MDC García-López H Cortés-Pérez I. Virtual reality-based interventions improve balance skills in children with developmental coordination disorder: systematic review and meta-analysis. Disabil Rehabil. (2025) 47:4617–28. doi: 10.1080/09638288.2025.2458186, 39876564

[26] Juras G Brachman A Michalska J Kamieniarz A Pawłowski M Hadamus A . Standards of virtual reality application in balance training programs in clinical practice: a systematic review. Games Health J. (2019) 8:101–11. doi: 10.1089/g4h.2018.0034, 30239217

[27] Green D Wilson PH. Use of virtual reality in rehabilitation of movement in children with hemiplegia − a multiple case study evaluation. Disabil Rehabil. (2012) 34:593–604. doi: 10.3109/09638288.2011.613520, 21978233

[28] Green D Lingam R Mattocks C Riddoch C Ness A Emond A. The risk of reduced physical activity in children with probable developmental coordination disorder: A prospective longitudinal study. Res Dev Disabil. (2011) 32:1332–42. doi: 10.1016/j.ridd.2011.01.040, 21334850

[29] Bian M Shen Y Huang Y Wu L Wang Y He S . A non-immersive virtual reality-based intervention to enhance lower-extremity motor function and gait in patients with subacute cerebral infarction: A pilot randomized controlled trial with 1-year follow-up. Front Neurol. (2022) 13:985700. doi: 10.3389/fneur.2022.985700, 36267888 PMC9577285

[30] Zheng P Yuan K Liu S Xue Z Ma P Teo EW . Effects of virtual reality technology on attention deficit in children with ADHD: a systematic review and meta-analysis. J Affect Disord. (2025) 384:127–34. doi: 10.1016/j.jad.2025.05.037, 40345442

[31] Jiang J Guo W Wang B. Effects of exergaming on executive function of older adults: a systematic review and meta-analysis. PeerJ. (2022) 10:e13194. doi: 10.7717/peerj.13194, 35433124 PMC9009327

[32] Sterne JAC Savović J Page MJ Elbers RG Blencowe NS Boutron I . RoB 2: A revised tool for assessing risk of bias in randomised trials. BMJ. (2019) 366:l4898. doi: 10.1136/bmj.l4898, 31462531

[33] Xie CX Machado GC. Clinimetrics: grading of recommendations, assessment, development and evaluation (GRADE). J Phys. (2021) 67:66–7. doi: 10.1016/j.jphys.2020.07.003, 32859566

[34] Meader N King K Llewellyn A Norman G Brown J Rodgers M . A checklist designed to aid consistency and reproducibility of GRADE assessments: development and pilot validation. Syst Rev. (2014) 3:82. doi: 10.1186/2046-4053-3-82, 25056145 PMC4124503

[35] Castellini G Bruschettini M Gianola S Gluud C Moja L. Assessing imprecision in cochrane systematic reviews: A comparison of GRADE and trial sequential analysis. Syst Rev. (2018) 7:110. doi: 10.1186/s13643-018-0770-1, 30055658 PMC6064621

[36] Cumpston M Li T Page MJ Chandler J Welch VA Higgins JP . Updated guidance for trusted systematic reviews: A new edition of the cochrane handbook for systematic reviews of interventions. Cochrane Database Syst Rev. (2019) 10:ED000142. doi: 10.1002/14651858.ED000142, 31643080 PMC10284251

[37] Hernandez-Martinez J Coñapi-Union B Canales-Canales S Perez-Carcamo J Sanchez-Sanchez J Sánchez M . Effects of plyometric jump training on physical performance in female soccer players across the competitive level: a systematic review with meta-analysis of randomized controlled trials. Front Physiol. (2025) 16:1675849. doi: 10.3389/fphys.2025.1675849, 41103293 PMC12521099

[38] Zhidong C Wang X Yin J Song D Chen Z. Effects of physical exercise on working memory in older adults: a systematic and meta-analytic review. Eur Rev Aging Phys Act. (2021) 18:18–33. doi: 10.1186/s11556-021-00272-y, 34535084 PMC8447686

[39] Perez-Carcamo J Hernandez-Martinez J Vásquez-Carrasco E Fernandez-Cardenas D Branco BHM Sandoval C . Effectiveness of non-immersive virtual reality on gross motor function, balance, and functional independence in children with cerebral palsy: a systematic review with meta-analysis. J Clin Med. (2025) 14:7582. doi: 10.3390/jcm14217582, 41226979 PMC12608441

[40] Wu J Zhang H Chen Z Fu R Yang H Zeng H . Benefits of virtual reality balance training for patients with parkinson disease: systematic review, meta-analysis, and meta-regression of a randomized controlled trial. JMIR Serious Games. (2022) 10:e30882. doi: 10.2196/30882, 35230242 PMC8924777

[41] Liu C Liang X Sit CHP. Physical activity and mental health in children and adolescents with neurodevelopmental disorders: a systematic review and meta-analysis. JAMA Pediatr. (2024) 178:247–57. doi: 10.1001/jamapediatrics.2023.6251, 38285440 PMC10825789

[42] Cavalcante Neto JL Steenbergen B Wilson P Zamunér AR Tudella E. Is wii-based motor training better than task-specific matched training for children with developmental coordination disorder? A randomized controlled trial. Disabil Rehabil. (2020) 42:2611–20. doi: 10.1080/09638288.2019.1572794, 30794762

[43] Bonney E Ferguson G Smits-Engelsman B. The efficacy of two activity-based interventions in adolescents with developmental coordination disorder. Res Dev Disabil. (2017) 71:223–36. doi: 10.1016/j.ridd.2017.10.013, 29055242

[44] Hammond J Jones V Hill EL Green D Male I. An investigation of the impact of regular use of the Wii fit to improve motor and psychosocial outcomes in children with movement difficulties: A pilot study. Child Care Health Dev. (2014) 40:165–75. doi: 10.1111/cch.12029, 23363371

[45] Mombarg R Jelsma D Hartman E. Effect of wii-intervention on balance of children with poor motor performance. Res Dev Disabil. (2013) 34:2996–3003. doi: 10.1016/j.ridd.2013.06.008, 23827983

[46] Harkness-Armstrong C Hodson-Tole E Wood G Mills R. Short report on a 6-week at-home exergaming intervention to improve balance in children with developmental coordination disorder. Res Dev Disabil. (2025) 156:104900. doi: 10.1016/j.ridd.2024.104900, 39700647

[47] Salem Y Gropack SJ Coffin D Godwin EM. Effectiveness of a low-cost virtual reality system for children with developmental delay: A preliminary randomised single-blind controlled trial. Physiotherapy. (2012) 98:189–95. doi: 10.1016/j.physio.2012.06.003, 22898574

[48] Yunus FT Widagda IM Isma R. The effect of sensory-motor virtual reality on balance in children with clinical down syndrome. Jurnal Kedokteran Diponegoro (Diponegoro Medical Journal). (2024) 13:66–71. doi: 10.14710/dmj.v13i2.42137

[49] Wang S Yu H Lu Z Wang J. Eight-week virtual reality training improves lower extremity muscle strength but not balance in adolescents with intellectual disability: A randomized controlled trial. Front Physiol. (2022) 13:1053065. doi: 10.3389/fphys.2022.1053065, 36483298 PMC9723224

[50] Wuang Y-P Chiang C-S Su C-Y Wang C-C. Effectiveness of virtual reality using wii gaming technology in children with down syndrome. Res Dev Disabil. (2011) 32:312–21. doi: 10.1016/j.ridd.2010.10.002, 21071171

[51] Rafiei Milajerdi H Sheikh M Najafabadi MG Saghaei B Naghdi N Dewey D. The effects of physical activity and exergaming on motor skills and executive functions in children with autism spectrum disorder. Games Health J. (2021) 10:33–42. doi: 10.1089/g4h.2019.0180, 33370161

[52] Kinney AR Eakman AM Graham JE. Novel effect size interpretation guidelines and an evaluation of statistical power in rehabilitation research. Arch Phys Med Rehabil. (2020) 101:2219–26. doi: 10.1016/j.apmr.2020.02.017, 32272106

[53] Levac D Rivard L Missiuna C. Defining the active ingredients of interactive computer play interventions for children with neuromotor impairments: A scoping review. Res Dev Disabil. (2012) 33:214–23. doi: 10.1016/j.ridd.2011.09.007, 22093667

[54] Nudo RJ Wise BM SiFuentes F Milliken GW. Neural substrates for the effects of rehabilitative training on motor recovery after ischemic infarct. Science. (1996) 272:1791–4. 8650578 10.1126/science.272.5269.1791

[55] Guadagnoli MA Lee TD. Challenge point: a framework for conceptualizing the effects of various practice conditions in motor learning. J Mot Behav. (2004) 36:212–24. doi: 10.3200/JMBR.36.2.212-224, 15130871

[56] Smits-Engelsman B Bonney E Ferguson G. Motor skill learning in children with and without developmental coordination disorder. Hum Mov Sci. (2020) 74:102687. doi: 10.1016/j.humov.2020.102687, 33017722

[57] Steenbergen B Van Der Kamp J Verneau M Jongbloed-Pereboom M Masters RSW. Implicit and explicit learning: applications from basic research to sports for individuals with impaired movement dynamics. Disabil Rehabil. (2010) 32:1509–16. doi: 10.3109/09638288.2010.497035, 20575752

[58] Mullen R Hardy L Oldham A. Implicit and explicit control of motor actions: revisiting some early evidence. Br J Psychol. (2007) 98:141–56. doi: 10.1348/000712606X114336, 17319055

[59] Wulf G Lewthwaite R. Optimizing performance through intrinsic motivation and attention for learning: the OPTIMAL theory of motor learning. Psychon Bull Rev. (2016) 23:1382–414. doi: 10.3758/s13423-015-0999-9, 26833314

[60] Drazich BF McPherson R Gorman EF Chan T Teleb J Galik E . In too deep? A systematic literature review of fully-immersive virtual reality and cybersickness among older adults. J Am Geriatr Soc. (2023) 71:3906–15. doi: 10.1111/jgs.18553, 37560978

[61] Sutcliffe TL Gaetz WC Logan WJ Cheyne DO Fehlings DL. Cortical reorganization after modified constraint-induced movement therapy in pediatric hemiplegic cerebral palsy. J Child Neurol. (2007) 22:1281–7. doi: 10.1177/0883073807307084, 18006957

[62] Taubert M Draganski B Anwander A Müller K Horstmann A Villringer A . Dynamic properties of human brain structure: learning-related changes in cortical areas and associated fiber connections. J Neurosci. (2010) 30:11670–7. doi: 10.1523/JNEUROSCI.2567-10.2010, 20810887 PMC6633410

[63] Levac DE Huber ME Sternad D. Learning and transfer of complex motor skills in virtual reality: a perspective review. J NeuroEngineering Rehabil. (2019) 16:121–36. doi: 10.1186/s12984-019-0587-8, 31627755 PMC6798491

[64] Li S Song Y Cai Z Zhang Q. Are active video games useful in the development of gross motor skills among non-typically developing children? A meta-analysis. BMC Sports Sci Med Rehabil. (2022) 14:140–55. doi: 10.1186/s13102-022-00532-z, 35870986 PMC9308223

[65] Lei Z Yuan K Xu J Miao Y Dai Y Wang J . Effects of physical exercises on balance in children with down syndrome: a systematic review and meta-analysis. BMC Sports Sci Med Rehabil. (2025) 17:165–76. doi: 10.1186/s13102-025-01222-2, 40604949 PMC12220196

