# Supplementary material for: Effect of virtual reality-based training on postural balance in children and adolescents with neurodevelopmental disorders: a systematic review and meta-analysis
Source: Front Public Health. 2026 Jan 9;13:1719286. doi: 10.3389/fpubh.2025.1719286 (PMC12827787; doi:10.3389/fpubh.2025.1719286)
Supplement: Supplementary file 1 [file Data_Sheet_1.pdf]

Pubmed

(child*[All Fields] OR preschool*[All Fields] OR adolescent*[All Fields] OR youth*[All Fields] OR teen*[All Fields]) AND ("neurodevelopmental disorder*"[All Fields] OR "child mental disorder*"[All Fields] OR "attention deficit hyperactivity disorder*"[All Fields] OR ADHD[All Fields] OR ADDH[All Fields] OR "attention deficit disorder*"[All Fields] OR "hyperkinetic syndrome"[All Fields] OR "minimal brain dysfunction"[All Fields] OR "autism spectrum disorder*"[All Fields] OR ASD[All Fields] OR autistic[All Fields] OR "developmental disabilit*"[All Fields] OR "developmental delay*"[All Fields] OR "child development disorder*"[All Fields] OR "developmental disorder*"[All Fields] OR "motor skills disorder*"[All Fields] OR "developmental coordination disorder*"[All Fields] OR DCD[All Fields] OR "intellectual disabilit*"[All Fields] OR ID[All Fields] OR "intellectual development disorder*"[All Fields] OR "mental retardation"[All Fields]) AND ("virtual reality exposure therapy"[All Fields] OR "virtual reality therapy"[All Fields] OR "virtual reality therapies"[All Fields] OR "virtual reality immersion therapy"[All Fields] OR "educational virtual reality"[All Fields] OR "educational virtual realities"[All Fields] OR "virtual reality exercise*"[All Fields] OR "virtual reality exercises"[All Fields] OR exergame*[All Fields] OR exergaming[All Fields] OR "active video gaming"[All Fields] OR "active-video gaming"[All Fields] AND ("postural control"[All Fields] OR "postural controls"[All Fields] OR "posture control"[All Fields] OR "posture controls"[All Fields] OR "postural equilibrium"[All Fields] OR "posture equilibrium"[All Fields] OR "posture balance"[All Fields] OR "posture balances"[All Fields] OR "musculoskeletal equilibrium"[All Fields] OR "motor skill*"[All Fields] OR balance[All Fields])

Web of science

TS=(child* OR preschool* OR adolescent* OR youth* OR teen*) OR ALL=(child* OR preschool* OR adolescent* OR youth* OR teen*) AND TS=("Neurodevelopmental disorder" OR "Attention Deficit Disorder with Hyperactivity" OR "Autism Spectrum Disorder" OR "Developmental Disabilities" OR "Motor Skills Disorders" OR "Intellectual Disability") AND TS=("Virtual Reality Exposure Therapy" OR "Virtual Reality" OR "Exergaming") OR ALL=("virtual reality exposure therapy" OR "virtual reality therapy" OR "virtual reality therapies" OR "virtual reality immersion therapy" OR "educational virtual reality" OR "educational virtual realities" OR "virtual reality exercise*" OR "virtual reality exercises" OR exergame* OR exergaming OR "active video gaming" OR "active-video gaming") AND ALL=("neurodevelopmental disorder*" OR "child mental disorder*" OR "attention deficit hyperactivity disorder*" OR ADHD OR ADDH OR "attention deficit disorder*" OR "hyperkinetic syndrome" OR "minimal brain dysfunction" OR "autism spectrum disorder*" OR ASD OR autistic OR "developmental disabilit*" OR "developmental delay*" OR "child development disorder*" OR "developmental disorder*" OR "motor skills disorder*" OR "developmental coordination disorder*" OR DCD OR "intellectual disabilit*" OR ID

OR "intellectual development disorder*" OR "mental retardation") AND ALL=("postural control" OR "postural controls" OR "posture control" OR "posture controls" OR "postural equilibrium" OR "posture equilibrium" OR "posture balance" OR "posture balances" OR "musculoskeletal equilibrium" OR "motor skill*" OR balance)

Scopus

TITLE-ABS-KEY(child* OR preschool* OR adolescent* OR youth* OR teen*) AND TITLE-ABS-KEY("neurodevelopmental disorder*" OR "child mental disorder*" OR "attention deficit hyperactivity disorder*" OR ADHD OR ADDH OR "attention deficit disorder*" OR "hyperkinetic syndrome" OR "minimal brain dysfunction" OR "autism spectrum disorder*" OR ASD OR autistic OR "developmental disabilit*" OR "developmental delay*" OR "child development disorder*" OR "developmental disorder*" OR "motor skills disorder*" OR "developmental coordination disorder*" OR DCD OR "intellectual disabilit*" OR ID OR "intellectual development disorder*" OR "mental retardation") AND TITLE-ABS-KEY("virtual reality exposure therapy" OR "virtual reality therapy" OR "virtual reality therapies" OR "virtual reality immersion therapy" OR "educational virtual reality" OR "educational virtual realities" OR "virtual reality exercise*" OR "virtual reality exercises" OR exergame* OR exergaming OR "active video gaming" OR "active-video gaming") AND TITLE-ABS-KEY("postural control" OR "postural controls" OR "posture control" OR "posture controls" OR "postural equilibrium" OR "posture equilibrium" OR "posture balance" OR "posture balances" OR "musculoskeletal equilibrium" OR "motor skill*" OR balance)

Cochrane Library

(child* OR preschool* OR adolescent* OR youth* OR teen*):ti,ab,kw AND ((neurodevelopmental NEXT disorder*) OR (child NEXT mental NEXT disorder*) OR (attention NEXT deficit NEXT hyperactivity NEXT disorder*) OR ADHD OR ADDH OR (attention NEXT deficit NEXT disorder*) OR "hyperkinetic syndrome" OR "minimal brain dysfunction") OR ((autism NEXT spectrum NEXT disorder*) OR ASD OR autistic OR (developmental NEXT disabilit*) OR (developmental NEXT delay*) OR (child NEXT development NEXT disorder*) OR (developmental NEXT disorder*)) OR ((motor NEXT skills NEXT disorder*) OR (developmental NEXT coordination NEXT disorder*) OR DCD OR (intellectual NEXT disabilit*) OR ID OR (intellectual NEXT development NEXT disorder*) OR "mental retardation")):ti,ab,kw AND ("virtual reality exposure therapy" OR "virtual reality therapy" OR "virtual reality therapies" OR "virtual reality immersion therapy") OR ("educational virtual reality" OR "educational virtual realities" OR ("virtual reality" NEXT exercise*) OR "virtual reality exercises") OR (exergame* OR exergaming OR "active video gaming" OR "active-video gaming"):ti,ab,kw AND ("postural control" OR "postural controls" OR "posture control" OR "posture controls") OR ("postural

equilibrium" OR "posture equilibrium" OR "posture balance" OR "posture balances" OR "musculoskeletal equilibrium") OR ((motor NEXT skill*) OR balance):ti,ab,kw

CNKI

TKA="儿童" +"学龄儿童" +"青少年" AND TKA="神经发育障碍" +"注意力缺陷多动障碍" +"ADHD" +"注意力缺陷障碍" +"多动症" +"智力障碍" +"智力低下"+"自闭症谱系障碍" +"自闭症" +"孤独症" +"ASD" +"发育障碍" +"发育迟缓" +"发育落后" +"发育残疾"+"智力发育障碍" +"智力发育迟缓" +"智力缺陷" +"认知发育障碍" AND TKA="虚拟现实" +"增强现实" +"混合现实" +"虚拟环境" +"虚拟现实技术" +"虚拟仿真" +"虚拟技术"+"VR" +"VR 技术" +"虚拟情景" +"沉浸式游戏" +"体感游戏" +"虚拟游戏" AND TKA="姿势平衡" +"平衡能力" +"下肢功能" +"姿势控制" +"平衡控制"+"平衡功能" +"运动技能" +"动作技能" +"运动能力" +"动作发展"

Wanfang

主题:("儿童" OR "学龄儿童" OR "青少年") AND 主题:("神经发育障碍" OR "注意力缺陷多动障碍" OR "ADHD" OR "注意力缺陷障碍" OR "多动症" OR "智力障碍" OR "智力低下" OR "自闭症谱系障碍" OR "自闭症" OR "孤独症" OR "ASD" OR "发育障碍" OR "发育迟缓" OR "发育落后" OR "发育残疾" OR "智力发育障碍" OR "智力发育迟缓" OR "智力缺陷" OR "认知发育障碍") AND 主题:("虚拟现实" OR "增强现实" OR "混合现实" OR "虚拟环境" OR "虚拟现实技术" OR "虚拟仿真" OR "虚拟技术" OR "VR" OR "VR 技术" OR "虚拟情景" OR "沉浸式游戏" OR "体感游戏" OR "虚拟游戏") AND 主题:("姿势平衡" OR "平衡能力" OR "下肢功能" OR "姿势控制" OR "平衡控制" OR "平衡功能" OR "运动技能" OR "动作技能" OR "运动能力" OR "动作发展")
